# Intelligent Supply Chain and Logistics Route Optimization Algorithm in Wireless Sensor Network

**DOI:** 10.1155/2022/8161820

**Published:** 2022-05-27

**Authors:** Zhijun Ma, Xiaobei Yang, Huahui Li

**Affiliations:** ^1^School of Management Engineering, Zhengzhou University of Aeronautics, Zhengzhou 450046, Henan, China; ^2^School of Tourism Management, Henan Finance University, Zhengzhou 450046, Henan, China; ^3^School of Management, Universiti Sains Malaysia, Minden 11800, Penang, Malaysia

## Abstract

This article first proposes the design of the ZigBee wireless sensor network node based on Arduino, from the design and implementation of the node hardware system to the design and implementation of the node software system, and expands on the design details. The node function was applied to the node design environment, and simulation tests were performed, and execution was tested to confirm the feasibility and practicability of the design. Based on this, the article proposes the service construction process of the software and hardware platform of the smart supply chain system, and then clarifies the basic mathematical problem of the distribution plan in line with the service strategy, that is, “the logistics route optimization algorithm with capacity and time constraint optimization,” which aims to study statically design, define, and model, and test two precise solutions representing heuristic genetic algorithms and analyze their results. In addition, additional hardware such as smart supply chain and cabinet boards are tested and designed according to system requirements, and the interaction mechanism is adapted to the user interface and supporting structure design so as to ensure the completion of user-related business processes. Combining the existing theoretical knowledge, the first priority is to meet customer needs, build a smart supply chain model under the premise of on-time delivery, and solve the model in time. Based on this point, this article tries to use hybrid branch algorithm link and tabu search algorithm to increase the consideration of traffic conditions, collect accurate algorithms, and modern heuristic algorithms to solve the actual situation of the case. At the end, the article proposes some related issues for the above problems. Suggesting through the research of wireless sensor network, this paper applies it to the research of intelligent supply chain and logistics route optimization algorithm and promotes the development of more intelligent supply chain in the future.

## 1. Introduction

This article is based on the design of the ZigBee wireless sensor node developed by Arduino, which provides a reference for the design and practical application of the wireless sensor network node. However, due to partial limitations of its time and capacity, this article has many shortcomings and deficiencies in the process of studying the ZigBee wireless sensor network node integrated with Arduino technology, so it is necessary to improve the function of the node. For example, consider combining this wireless sensor network node with the existing 3G/4G mobile network to achieve greater network coverage in terms of regional monitoring and multilevel access [1]. Wireless sensor network has very good application prospects. It is believed that with the deepening and progress of research, wireless sensor network will be able to better serve people's production and life [2]. Correspondingly, the article clarifies that a certain smart supply chain model defined has a specific feasibility, and through reading related literature and research papers to realize that the designed algorithm can solve related problems to a certain extent and have certain optimization results [3]. The service and control platform based on this smart supply chain model has the possibility and value of in-depth research and application. Compared with the current smart supply chain model, the related processes and user experience are a new attempt [4]. Finally, this paper studies the optimization algorithm of logistics routes [5]. In the logistics industry, distribution is at a key position, and it plays an important role in the entire system. The logistics industry is also developing in the direction of globalization, informatization, and integration [6]. In a highly developed business society, due to the competition between industries and the stricter requirements of consumers for delivery time, logistics companies pay more attention to information science and efficient management of logistics and distribution operations. Whether the transportation and distribution routes are reasonable has a significant impact on the speed, cost and efficiency of distribution [7]. The optimization of logistics distribution channels can provide guarantee for the scientific management of enterprise logistics, thereby improving the efficiency of logistics cost operation and increasing the capital turnover rate [8]. Therefore, studying this problem can help logistics companies provide high-quality services to customers and improve the company's service quality level to ensure its stable operation under contemporary production models [9]. The most important measures taken by the research are to shorten the business operation cycle, save various transportation costs, thereby reduce overall logistics costs, improve the efficiency of vehicle transportation, optimize, and fair allocation of resources so as to achieve checks and balances in protecting the environment and saving social resources and show its importance [10]. Therefore, how to reduce the transportation cost of logistics distribution, find the fastest logistics route, and propose an optimization algorithm is an urgent and important research topic at this stage [11].

## 2. Related Work

The literature introduces the research background and importance of this research topic, then introduces the current research status of this topic at home and abroad, and finally briefly introduces the main research content and thesis writing structure of this article [12]. The literature introduces the hardware design scheme of ZigBee wireless sensor network node based on Arduino including the overall structure of the hardware node design framework, the hardware design for each applicable wireless sensor network node module, the PCB design involved in the wireless sensor network hardware node design process, and the physical object of the wireless sensor is the network system formed in the plan [13]. The literature introduces the software design scheme of ZigBee wireless sensor node based on Arduino. According to the composition of the wireless sensor network software system, analyze the design and research of the communication node, analyze the wireless network node software system and various components used including the main design process of the node, collect sensor data, check battery power information and other design and execution procedures, and the design and implementation of the host computer software, and expanded the system specification of the node software in detail [14, 15]. The literature describes the delivery scheme and user interface of the delivery vehicle. It mainly introduces the software design and hardware functions of the vehicle, as well as the logic used in the user interface [16]. It aims to realize the related service process and provide a reference for the actual development and application of solutions such as parameters and processes. The literature introduces the function and test of ZigBee wireless sensor node based on Arduino [17]. First, the three-node communication function between the node and the computer, the node communication function of the point-to-point communication mode, and the sensor node data collection function were tested. On this basis, the network test of the sensor node and the function test of the wireless sensor network system after the network maintenance are carried out. Finally, the power consumption of the node was tested and analyzed, and compared with the power consumption of the node of other technologies [18, 19].

## 3. Wireless Sensor Network and Logistics Route Optimization Algorithm

### 3.1. Wireless Sensor Network

A wireless sensor network can be defined as a wireless sensor network is composed of many low-cost and small sensor nodes, which form a multihop, self-organizing system in the network through the wireless transmission of information [20]. The main purpose of the wireless sensor network is to collect and process the information to be monitored in the monitoring area, and to process the collected monitoring results and provide them to users. It can be seen from the definition and purpose of wireless sensor networks that sensors, monitoring targets and users are the basic elements of wireless sensor networks. The wireless network is the key channel connecting the main elements [21]. The user switches the sensor network through wireless communication and monitors sensor information in a collaborative manner. The characteristics of wireless sensor networks include limited power supply capacity, limited storage and computer capacity, limited information transmission capacity, and a large number of nodes scattered in multiple locations [22].

**Figure 1:**
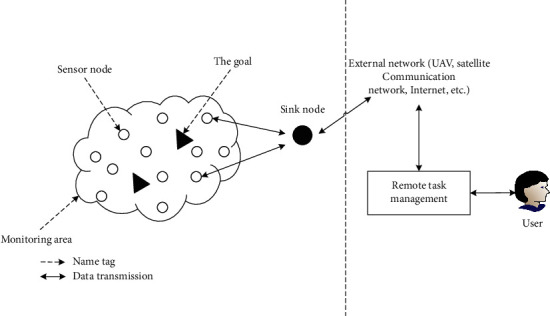
Structure diagram of wireless sensor network system.

The structure of the wireless sensor network system is shown in Figure 1. Generally speaking, the nodes that make up a wireless sensor network can be divided into three different nodes according to their functions, namely, sensor nodes, sink nodes, and management nodes. The working process of the wireless sensor network system can be divided into the sensor data transmission direction and the wireless sensor network control direction according to the data flow direction. Among them, in the working process, the direction of data transmission to the sensor is the sensor nodes scattered in the monitoring area can be used as terminal nodes to collect sensor information, or as transfer nodes. The sensor information captured by single-hop or multihop terminal nodes is transmitted to the sink node through this method, and then sent to external networks such as drones, satellite communications, or the Internet. The data information will be transmitted to the remote task management node through the external network so that the remote task management node processes the received data first. After the user receives the data from the remote task management node, he can process the data according to his needs. The process of controlling the wireless sensor network is the user directly uses the remote activity management node to monitor, sends the wireless sensor network control information to the remote activity management node, and then sends the task control information of the remote management node to the external network. The control information is then sent from the external network to the sink node and then transmitted from the sink node to the sensor node.

The API identifier in the API serial data frame describes the type of API information contained in the following identification data. If the XBee ZB module performs wireless communication under the API serial port protocol, the module obtains the main purpose and content of the information frame after determining the API identifier type of the API data serial frame. Start the next action according to the content of the message box. Some API identifiers defined in XBee ZB and the API frame name corresponding to each identifier is shown in Table 1.

The API ID corresponding to the AT control frame is 0 × 08. Enabling this type of API framework involves querying or setting module parameters on the local device. The XBee ZB module defines a total of 93 AT commands, and different AT commands correspond to different parameters or actions. Using the AT control frame to change the parameters of the XBee ZB module will take effect immediately. The data area structure of the AT control frame is shown in Table 2.

The analog sensor chosen in this article is the LM35 analog temperature sensor. LM35 is a low-power integrated circuit temperature measurement device, the temperature measurement range is between −55°C−150°C, and the measurement error is 0.5°C (outdoor temperature is 25°C). The operating voltage of LM35 is between 4 V–30 V, the current loss is less than 60uA, and the output impedance is low. For a load whose output is 1 mA, its output impedance is 0.1 Ω. The linear voltage output of LM35 is proportional to the temperature in degrees Celsius. The proportional formula is shown as

(1) VOUT=10mvFT∗T.

According to the characteristics of LM35 and the functions of Arduino, the calculation formula for the ambient temperature of Arduino measured by LM35 can be obtained as

(2) $$\begin{array}{c} T=5.0^{*}\text{analog Read}{\left(Pin\right)}^{*}\frac{100.0}{1024}. \end{array}$$

The formula for calculating the remaining battery capacity according to the net value of battery energy accumulated in the ICA register is

(3) $$\begin{array}{c} {\text{CAP}}_{RE}=\frac{\text{ICA}}{\left(2048^{*}R_{\text{SENS}}\right)}. \end{array}$$

### 3.2. Establishment of Optimization Model

Since the solution tasks of each delivery time are independent of each other and the problem types are the same, it is suitable for the same set of solution models. The following modeling involves the internal conditions at the time of delivery.

The total number of vehicles *k* can be the “maximum waiting time of service” provided for a given delivery task. The *k* value is related to the distribution service strategy and the total number of vehicles transported. Among them, it is assumed that each transportation waits for 5 minutes.

(4) $$\begin{array}{c} t_{k}=5*\sum_{\begin{array}{c} i\in N,j\in N\\ i\neq j \end{array}}X_{ij}^{k}\ldots \ldots \forall k\in V. \end{array}$$

The judgment change is defined as “if the vehicle moves from one point *i* to another point *j*.” The relevant constraints given in formula (5) can ensure that once this process occurs, the quantity of goods to be sent at point *j* is not zero.

(5) $$\begin{array}{c} X_{ij}^{k}=\left\{\begin{array}{c} 1\text{ car }k\text{ from }i\;to\;j;\\ 0\text{ car cannot }k\text{ from }i\text{ to }j; \end{array}\right). \end{array}$$

The main goal is to reduce the total mileage of all vehicles in each phase of the delivery task as follows:

(6) $$\begin{array}{c} \sum_{k\in V}\sum_{i\in N,i\neq j}X_{ij}^{k}c_{ij}^{k}=d_{j}\ldots \ldots \forall j\in N,\\ X_{ij}^{k}\leq c_{ij}^{k}\ldots \ldots \forall k\in V,\forall i\in N,\forall j\in N,i\neq j,\\ \sum_{\begin{array}{c} i\in N,j\in N\\ i\neq j \end{array}}c_{ij}^{k}\leq Q\ldots \ldots \forall k\in V,\\ X_{ij}^{k}\geq X_{ij}^{k+1}\ldots \ldots \forall k\in V\setminus \left\{v\right\},\forall i\in N,\forall j\in N,i\neq j,\\ \sum_{i\in N,i\neq m}X_{im}^{k}=\sum_{j\in N,j\neq m}X_{mj}^{k}\ldots \ldots .\forall k\in V,\forall m\in N\setminus \left\{1\right\},\\ \sum_{\begin{array}{c} i\in N,j\in N\\ i\neq j \end{array}}\frac{X_{ij}^{k}D_{ij}}{\bar{v}+t_{k}}\leq 120\ldots \ldots \forall k\in V,\\ X_{ij}^{k}\in \left\{0,1\right\}\ldots \ldots \forall k\in V,\forall i\in N,\forall j\in N,i\neq j. \end{array}$$

The resulting solution will include a route plan for each vehicle (including the order of points requested on the route and the number of deliveries at each point) as well as the number of vehicles that follow and then calculate the time spent by each vehicle for trial use reference.

The simplest construction of the VRP model is a delivery point, which delivers items to multiple demand points as needed and finds the shortest transportation route. In order to make the model theory feasible, this article needs to add some assumptions as follows.

Vehicle fixed cost, that is, the fixed cost required for each vehicle to be shipped, and the total fixed cost:

(7) $$\begin{array}{c} C_{g}=\sum_{k=0}^{V}cg_{k}. \end{array}$$

The cost of vehicle transportation is directly proportional to its transportation distance and the total cost of transportation.

(8) $$\begin{array}{c} C_{y}=\sum_{k=1}^{v}\sum_{i=1}^{n}\sum_{j=1}^{n}C_{ij}d_{ij}X_{ij}. \end{array}$$

All delivery vehicles depart from the distribution center and finally return to the distribution center:

(9) $$\begin{array}{c} \sum_{i=0}^{k}y_{ik}=\left\{\begin{array}{cc} 1, & i=1,2,\ldots n\\ k, & i=0 \end{array}\right). \end{array}$$

Also, it must meet the requirements of each customer and can only be delivered by a delivery vehicle.

(10) $$\begin{array}{c} \sum_{k=1}^{v}\sum_{j=1}^{n}x_{ijk}=1. \end{array}$$

Based on the above analysis, the following model can be constructed:

(11) $$\begin{array}{c} \text{Min}=C_{k}+C_{y}\\ =\sum_{k=0}^{v}cg_{k}+\sum_{k=1}^{v}\sum_{i=1}^{n}\sum_{j=1}^{n}C_{ij}d_{ij}X_{ij},\\ \text{St}.\sum_{i=0}^{k}i=0=\left\{\begin{array}{cc} 1, & i=1,2,\ldots n\\ k, & i=0 \end{array}\right.,\\ \sum_{k=1}^{v}\sum_{j=1}^{n}x_{ijk}=1,\\ \begin{array}{c} E\leq t_{ij}\leq L\\ X_{ijk}=0\text{ or }1\;i,j=1,2\ldots \ldots N \end{array}. \end{array}$$

Delivery and transportation with time constraints were modeled, such as urgent delivery time requirements, special needs of some users when stocks are out of stock, JIT production lines, delivery of trains, and scheduled airplanes. Also, the delivery requirements of the supermarket stipulate that before the door opens for business, and much later than the sale of goods is used up, etc. When constructing this model, this article still uses the VRP hypothetical model.

Vehicle fixed cost, fixed cost required for each vehicle shipment, total fixed cost:

(12) $$\begin{array}{c} C_{g}=\sum^{v}cg_{k}. \end{array}$$

The cost of transportation is directly proportional to the distance of the car and the total cost of transportation.

(13) $$\begin{array}{c} C_{y}=\sum_{k=1}^{v}\sum_{i=1}^{n}\sum_{j=1}^{n}C_{ij}d_{ij}X_{ij}. \end{array}$$

### 3.3. Model Solution Design

That is, the average demand of each building, through the “rounding” operation to clarify its interval, the maximum amount between each time period is recorded as

(14) $$\begin{array}{c} d_{\text{scale}}=\left[\frac{\max\left(d_{i}\right)}{10}\right)*10\ldots \ldots \forall i\in N. \end{array}$$

That is, the distance between the furthest demand point and the starting point of the distribution is equal to its number in the sequence through a “rounding” operation recorded as

(15) $$\begin{array}{c} D_{\text{scale}}=\left[\frac{\max\left(D_{ij}\right)}{50}\right)*5\ldots \ldots \forall i,j\in N,i\neq j. \end{array}$$

Consistent with actual needs, the scope of various factors that will affect the size of the introductory question is shown in Table 3.

If the size of the problem is specified, one value is subtracted from the three high angles. For example, the required number of points described in (4.20.150) is 4, the maximum average service scale is 20, and the maximum service distance does not exceed 150m. In the following, select any combination of parameters from small to large in Table 3 and simulate the needs of each point and the distance between the points and the cities, according to the scale.

In order to find out whether there is a suitable algorithm for solving small-scale problems, this section first establishes the corresponding overall jump model of the program according to the above mathematical model, and uses the Cplex solver to solve it.

Select the scale (n, *dscale*, *Dscale*) to (4.10.100), set the requirements between 1 and 10, and randomly generate each point, as shown in Table 4.

The coordination value of the demand point only works when the destination is clear. When calculating according to the actual road network information, the distance between points should be subject to actual monitoring and distance mapping.

In the actual design of the algorithm, it is found that if the combination and assignment process of roles is regarded as a CMVRP problem, the known conditions are few, the output demand is large, and the solution is complicated.

**Figure 2:**
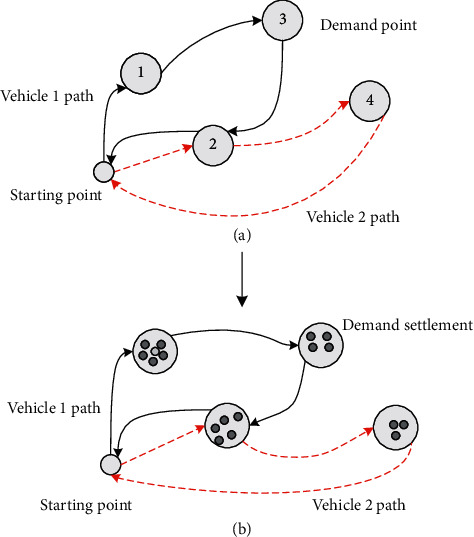
Genetic algorithm solution process design.

As shown in Figure 2, for each demand point with a set of known coordinate values and the distance between points, a non-negative number is required; each settlement point represents a demand, and the number of new “demand points” can be used as the initial demand point value. As a result, the problem can be reduced to an MTSP problem (see Figure 2), and the solution becomes simpler and faster after the simplification.

### 3.4. Simulation Experiment

**Figure 3:**
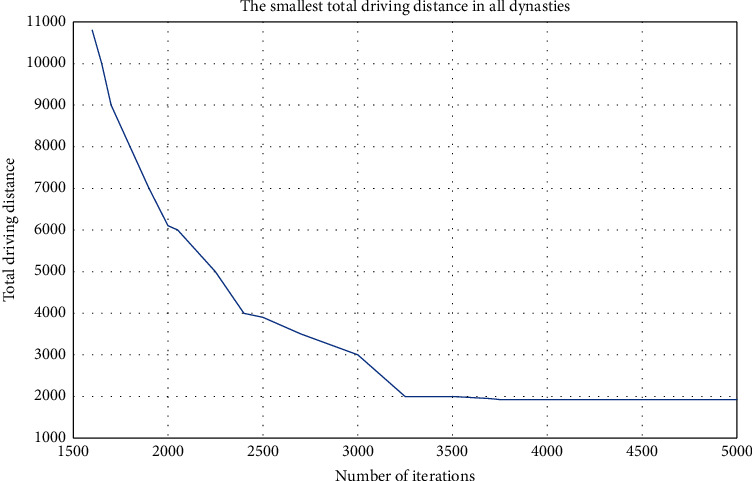
The convergence process of case solving optimization (number of vehicles: 10, number of runs: the third time).

**Figure 4:**
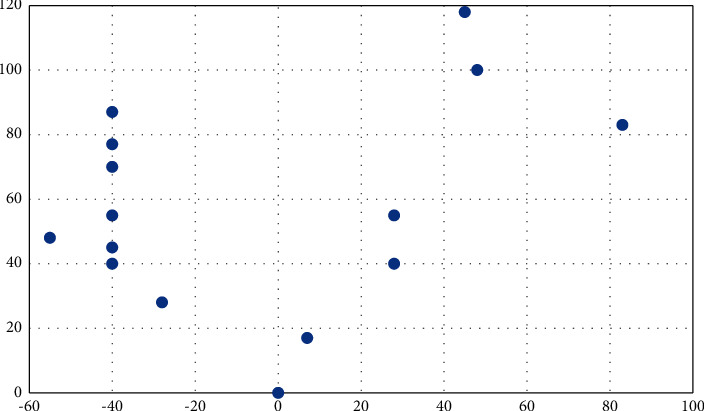
Case solution route plan (number of vehicles: 10, number of runs: the third time).

As shown in Figures 3 and 4, it is one of the optimization processes and the roadmap of the best solution. Combining several solution processes, it can be seen that the 5000-generation iterative process is sufficient to make the target volume reach a relatively stable level, and the processing optimization gain range is also relatively wide.

The planning route of the case solution is shown in Figure 4:

## 4. Research on the Design and Application of Smart Supply Chain Management Platform

### 4.1. System Frame Design

The intelligent supply chain logistics distribution platform software and hardware are combined to provide synchronization functions. This section designs the system framework from two aspects: hardware integration scheme and software platform, laying a foundation for the progress of subsequent research work.

**Figure 5:**
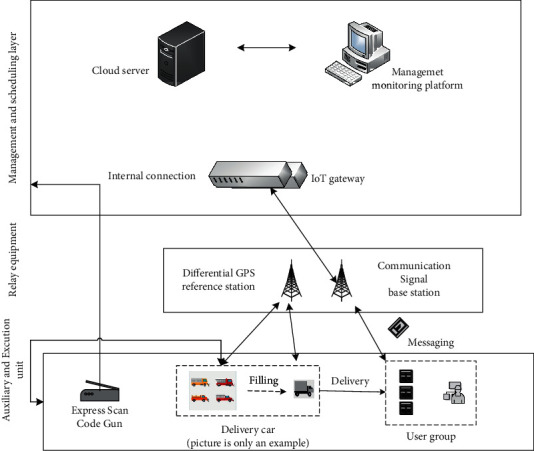
Hardware system design of unmanned distribution platform.

The hardware level is shown in Figure 5, mainly including responsible for storing data, issuing instructions, running algorithms, and monitoring resources.

When the express mail arrives at the delivery stations of various e-commerce and logistics companies, it is first scanned and input, collected and classified by processing algorithms, and then preprocessed, and the vehicle information is restored through the solving algorithm, and then the program is released and the express information is loaded to the vehicle. Tasks in execution, at the same time release the actual vehicle operation control instructions, and cooperate with GPS navigation to guide the vehicle to drive along the designated route, and various GPS navigation equipment signals such as signals and areas are sent during transportation.

It is responsible for correcting navigation signals and transmitting wireless communication signals.

The coverage of a single GPS station is hundreds of kilometers. For smaller urban areas, a station can be set up in the central area without special buildings. It can receive and send directly to the vehicle's satellite signals and location information, and can combine the deviation to determine the vehicle's driving direction and correct and guide it. The navigation error is reduced from 5m to centimeter level.

The basic signal communication station mainly provides relay services for the wireless communication process of the whole system to ensure good vehicle communication signals and avoid problems such as control failures and communication delays.

It is responsible for entering courier information, executing the delivery plan, and replying to users.

For express mails from various third-party logistics companies, efficient algorithms are required to capture and summarize their basic quality information, and prepare them in accordance with the rules and principles of mail processing.

After obtaining the delivery plan of the vehicle, the staff first appoints the plan to complete the declared delivery, and starts the machine when the delivery reaches the number set for the delivery. The vehicle has its own navigation module and relay equipment, integrates the information previously stored in the regional road network, and drives along the defined route.

As the last link in the process of providing intelligent services to the logistics distribution platform, the user terminal can perform interactive operations, such as explaining requirements and confirming receipts on the corresponding page of the smart phone.

Provide software support to ensure the correct operation of hardware devices including navigation, obstacle avoidance, communication, prevention, monitoring, and other modules. Its content must be continuously combined with the specific requirements of the algorithm logic design to ensure good cooperation between the units and realize the overall function.

Considering the specific mathematical problems identified, establishing mathematical models, designing, developing and verifying solving algorithms, and guiding the allocation and management of three consistent solutions in the operations are obtained.

### 4.2. Process Design of Smart Supply Chain

According to the business process and the information exchange process, we must fully consider the entire system process and the detailed problems that may arise from it, and continue to conduct detailed analysis and process design. The process design content in this section will lay the foundation for the service model of the entire unmanned distribution platform and guide further research.

The entire distribution process relies on a large amount of fixed information (such as regional geographic information, related service rules, procedures, etc.) to dynamically process, analyze, send, and deliver express mail. Also, share information resources, obtain as many or slightly better comprehensive distribution plans as possible, and combine the deployment of equipment (unmanned delivery vehicles, local navigation networks, servers, etc.) to achieve offline small-batch manual delivery. Among them, the accuracy of the obtained solution depends on the size of the problem, and the size of the problem depends on the upper limit of the ability to solve the appropriate algorithm.

In the initial construction of related service areas, the geographic information of the electronic zone usually consists of demand point coordinate information and road network information; if regional geographic information is generated, it will be saved and used together with related data variables as the main reference for determining the content.

In addition, the most important basis for decision-making is the system service method and related information processing mechanism. It mainly specifies the principles of mail processing, service mechanisms, analysis principles, algorithms, dynamic decision-making mechanisms, etc., serving the preliminary processing and dynamic distribution process of express information, the design of solutions, and the rapid response to the real offline distribution process.

In the distribution process itself, it is difficult to achieve the ideal state that the distribution task can be successfully completed every time. With the real-time delivery service, users often cannot pick up the goods on time due to different situations. Also, unmanned vehicles in small cities may also face some emergencies that affect normal delivery.

In this section, this article will outline appropriate decision-making mechanisms for these two special situations with multiple possibilities.

If there is a large amount of vehicle resources required to track the shipment load in the current time period or the tracking of the shipment load on the day, the vehicle will not wait for the user to time out and the machine is set to continue to track the delivery activity at the end time. At the same time, users are required to select a new delivery time.

If parking stagnation occurs, it may cause users to miss the delivery process at their designated time, and decision-making will be reduced.

Whenever the delivery is delayed, the user's delivery information will be imported into the active list, and the delivery number will remain unchanged. The algorithm can also solve all newly formed sequential delivery tasks to obtain a corrected delivery plan.

Under unsatisfactory circumstances, each batch of goods may not always be delivered in full within a period of time, and special circumstances and other special accidents may occur. At this point, the service process will be expanded according to the basic service process and dynamic decision-making mechanism.

In the allocation process, whenever a special situation is encountered, the evaluation mechanism is used to determine which situation is the first and initiate the corresponding decision-making process. If all special circumstances are fully covered, the final decision is to collect the goods next time.

### 4.3. Functional Module Design

The integrated platform adopts a multimodule collaboration method to realize rapid mail information input and output (formulation), communication, and implementation of delivery plans. The key feature is the algorithm tool used to calculate the mail distribution plan and vehicle delivery route, and the offline deployment unit and user interface composed of unmanned delivery vehicle equipment, etc.

Among them, the “task planning and scheduling algorithm” is the key link of the solution in the entire platform deployment plan. The data source is mainly part of the “user,” including most of the initial information to be transmitted and a small amount of information transmitted through “non-offline facilities.” It is also the main information that guides the hardware to perform delivery operations.

As the service target of the whole system, the “user” component not only plays an important role in the core development process but also guides various decision-making plans and the development process itself including interactive man-machine docking process and offline pickup.

“Offline facilities and equipment” include unloading and distribution stations and distribution vehicles. As the main hardware executor of the decision plan, the actual driving instructions are determined by the decision plan and road network area information. Real-time operating instructions come from the “Network and Server” navigation module.

“Network and server,” as the data mode connecting each unit, undertakes most of the information transmission tasks, and is also responsible for the storage, positioning, browsing, monitoring, and future learning activities of more qualified data.

The functional division and data interaction in each module will provide guidance for the design of subsequent related mechanisms and the development of software and hardware platforms.

Analyze the logistics process of intelligent supply management based on the distribution platform, data information, and equipment sources to investigate the resource management process of the entire system. The express mail information arrives at the “temporary database” after the initial collection, and combines the preprocessing scheme to perform simple clustering and time domain division; the processing result is transmitted to the “algorithm platform” as input information to obtain the delivery route of each vehicle. The staff completes the transportation according to the established plan and enters the corresponding vehicle transportation information in the “sales database.” The latter associates the attributes of the goods with the vehicle number and the corresponding internal number. Call to send notification information; the user takes out the package, all transportation information is stored in the “historical database.”

Based on the clear data flow, in order to facilitate the system to control the overall dynamics, perform appropriate operations or use it as a reference judgment, and it is also necessary to effectively manage various information of resources.

This section will integrate the system process design, introduce in detail the user intervention process in the supply chain distribution platform, design a user interface that can meet related needs, and provide a reference for the actual development of the platform.

In practice, the entire interaction and collaboration between the user and the vehicle should be mediated by the processing and response of the server. For a vehicle, its status must be reported at any time, including location information, user interaction information, body condition, and load. The latter has a preresponse mechanism to ensure specific operations under specific conditions.

When users perform operations such as querying and issuing instructions, they can access the corresponding database. The server mixes the data with the device status information and returns the results or notifications to the user. All log sorting operations, including successful and unsuccessful shipment information, are stored in the historical database. If the shipment is not completed, you can view and link to the corresponding operation page.

The delivery information of the user before the formal delivery will affect the actual execution process of the next day's delivery task. The confirmation information when an emergency occurs during the delivery process requires the participation of the user to implement an appropriate decision-making mechanism. If the user is not present, the delivery vehicle will also select the next destination and send a notification message to the user.

From a technical point of view, the user interface can be implemented through platforms or tools such as WeChat applets, official accounts, and apps, and can be used as the customer part of the intelligent supply of the entire supply chain logistics platform.

## 5. Conclusion

The article first introduces the Arduino-based wireless network node software system based on the ZigBee sensor node, the network node hardware system and sensor network node communication framework, including the design of the node software system and the upper computer design of the connection node software design. The software system design is divided into three parts, including the design of sensor node temperature and progress collection function, battery-powered information collection function and wireless data transmission function; graphical representation of the network function and wireless data reception function of the aggregation node, and the design of high-end computer data processing and data display functions. Finally, this article first introduces the different theories of logistics distribution, and then conducts part of the research on the optimization of logistics distribution routes. The optimization of the logistics distribution route belongs to the NP problem. Then, based on the analysis of the general automobile problem, the road conditions are added to make the VRP problem more difficult. This model takes into account the uncertain reasons of the road conditions, is close to the reality, and reduces the waiting time on the transportation route. A hybrid algorithm based on the branch link method and the tabu search algorithm is proposed to solve the logistics distribution route problem. The actual calculation example based on the time constraint shows that the algorithm is the best solution to prove.

## Figures and Tables

**Table 1:** Part of API identifiers and their corresponding API structure names.

API identifier	API frame name
0 × 08	AT command
0 × 10	ZigBee transmission request
0 × 17	Remote command request
0 × 88	AT command response
0 × 8A	Module status
0 × 8B	ZigBee transmission status
0 × 90	ZigBee data reception (AO = 0)
0 × 97	Remote command response

**Table 2:** The structure table of the identification data in the AT command frame.

Frame data element name	Description
API identifier	= 0 × 08, it means this frame is an AT command frame.
Frame ID	The confirmation is associated with a subsequent confirmation data to the host's serial data frame. If it is set to “0,” no response will be sent.
AT command	AT commands are composed of two ASCII characters. XBee has 93 AT commands. Different AT commands correspond to different parameters or actions.
Parameter value	The parameter value part is not a required structure. If there is a parameter value, this part should have data. If there is no parameter value, this part is not required.

**Table 3:** The main research scope of different elements.

N/piece	Dscale/piece	Dscale/m
3	10	50
4	20	100
5	30	1 50
6	40	200
7	50	250
8	60	300
9	70	350
10	80	400
11	90	450
12	100	500
13	110	550
14	120	600
15	130	650

**Table 4:** Demand at each point (example 1).

Demand point	Demand/piece
1 (starting point)	0
2	6
3	5
4	4
5	3

## Data Availability

The data used to support the findings of this study are available from the corresponding author upon request.
